# Effect of high-speed steel screw drill geometry on cutting performance when machining austenitic stainless steel

**DOI:** 10.1038/s41598-023-36448-y

**Published:** 2023-06-07

**Authors:** Josef Sedlak, Jan Zouhar, Stepan Kolomy, Martin Slany, Emil Necesanek

**Affiliations:** 1 Faculty of Mechanical Engineering, Brno University of Technology, Brno, Czech Republic; 2 NÁSTROJE CZ, s.r.o., Kyjov, Czech Republic

**Keywords:** Aerospace engineering, Mechanical engineering

## Abstract

Drilling into the solid material is one of the basic technological operations, which creates a cylindrical hole in an appropriate time with required quality. Drilling operation demands a favourable removal of chips from the cutting area because a creation of an undesirable shape of chips can impart a lower quality of the drilled hole corresponding with the generation of excess heat due to the intense contact of the chip with drill. The solution for a proper machining is a suitable modification of the drill geometry i.e., point and clearance angles as presented in current study. The tested drills are made of M35 high-speed steel characterized by a very thin core at the point of the drill. An interesting feature of the drills is the use of cutting speed higher than 30 m min^−1^, with the feed of 0.2 mm per revolution. The surface roughness (Ra and Rz lower than 1 µm and 6 µm respectively), cylindricity (0.045 mm), roundness (0.025 mm), perpendicularity of the hole axis (0.025 mm), diameters and position of the individual holes were achieved for a drill with point angle 138.32°and clearance angle 6.92 respectively. The increase of the drill point angle by 6° resulted in the decrease in the feed force of more than 150 N. In addition, an increase of the clearance angle by 1° resulted with a decrease in the feed force of 70 N. The results of the experiment showed that with the correct geometry of the tool the effective machining without using internal cooling can be realised.

## Introduction

Stainless steels are used in a wide range of industries due to their exceptional properties in the field of resistance to the external environment and elevated temperature. This steel can be used as surgical instruments in medicine, components for the exhaust system of cars or large reservoirs and pipelines in the petrochemical industry.

When drilling stainless steel, several limitations can act on the tool. These limitations include low thermal conductivity, high ductility, susceptibility to strain hardening and high abrasiveness of the machined material. Dolinšek^1^ examined strain hardening during drilling of austenitic stainless steel. With the help of the cut breaker, it was found that the highest intensity of deformation hardening occurred in the vicinity of the axis of the drill close to the point of the cross cutting edge. The result of strain hardening was that the microhardness of the material increased from 300 to 600 HV 50. Austenitic stainless steel is characterized by up to four times lower thermal conductivity compared to low-carbon steel, which causes an intense increase in temperature at the edge of the cutting tool^2^. The highest heat occurs on the periphery of the tool in the area of the outer point due to the increasing cutting speed outwards to the drill perimeter^3^. According to the simulation based on the experiment in the study^3^ performed on AISI 1045 carbon steel, almost 50% of the generated heat transferred into the tool. The intensity of heat generation can be reduced by decreasing the area of the inefficiently machining core of the drill, as in the case of the CZ005 tool from this study, or by using the appropriate clearance angle of the drill^4^. Effective cooling of the tool edge is another option for eliminating the generated heat. In the study^4^, the effect of internal cooling with the pressure of 80 bar towards to the cutting edge was analysed experimentally and through the simulation. It was found that, despite the high speed of liquid supply through the cooling channels, inefficient cooling of the tool edge occurred due to an undesirable fluid turbulence and friction on the drill. According to the results of a study^5^ conducted on austenitic stainless steel, an increase in the value of the point angle in the range of 82°–118° led to the increase in the temperature at the cutting edge. Under certain conditions, there may be a reduction in cutting forces due to the so-called softening effect^6^ leading to an improvement of surface roughness. The effect of the cutting speed on the roughness of the machined surface depends on the temperature at which it occurs to prevent the formation of growth in the given material, the values of the cutting speed used and the machined material. In the study^7^, the lower surface roughness of austenitic stainless steel (about 20–50%) was observed when the cutting speed increased from 18 to 30 m min^−1^. On the contrary, in the study^8^ when drilling low-alloy steel, there was an increase in roughness more than 50% when the cutting speed increased from 32 to 51 m min^−1^. The softening effect featured a major impact in the experiment used by Huang et al.^9^. They analysed the influence of individual parameters on the magnitude of the feed force when drilling thin plates made of composite material reinforced with silicon carbides. Increasing the cutting speed from 50 m min^−1^ to the double value featured a different effect on the amount of feed force at different stages of drilling. Before entrance of the entire tool tip, there was a slight increase in feed force due to the increase of cutting speed. In case of drilling workpiece with a thickness higher than 1 mm, when the cutting speed increased, the temperature increased and, but due to the softening effect, the feed force decreased by more than 15%.

The difficult conditions of drilling austenitic stainless steel described in the previous paragraph require the correct selection of individual geometric parameters of the tool, otherwise the rapid wear is observed. The tool wear is accompanied with the lower quality of the hole corresponding with the increase of the temperature and the high toughness of the material. From the complex geometry of the helical drill, the influence of the point angle on the cutting process is most often discussed in the studies. The first parameter analysed is the feed force. The dependence of the size of the point angle on the feed force varies in each study. It was found that when the point angle increased, the feed force slightly decreased^10,11^. The same trend was observed in the current study. On the other hand, the increase of the feed force when the point angle increased was presented by the other researchers^6,12,13^. When the point angle increased from 82° to 118° in the study^5^ and 110° to 118° in^14^ the intense deformation of the material at the point of the cross edge caused the higher temperature. Karpat^6^ performed a development of the special drills with a double edge aimed for machining composite materials. The drills featured the diameter of 6.4 mm, while the length of each drill varied as well as the length of the cutting edge, which enabled to change the dependence of the feed force on time. The drill with a lower point angle exhibited lower feed force compared to the drill with the higher point angle. They found out that the best results were achieved when the drill had the longest cutting edge with a point angle of 120°^6^. The lower feed force can decrease the delamination of the composite layers, or the formation of burrs when drilling ductile metal materials. Murad et al.^15^ presented the paper evaluating an effect of point angle when drilling of the titanium Ti6Al4V alloy. Due to the temperature increase in the hole surface, the hole featured the hardening layer. The higher point angle caused an increase in the thickness of the hardened layer of the hole from 0.05 to 0.1 mm. The increase in layer thickness was also observed when tool wear increased.

When machining tough stainless steel, long helical chips, which causes abrasive effect in the drill groove and in the drilled hole are formed. Due to the friction between the chip and the tool, an excess heat is generated, and the surface of the drilled hole may be damaged^3^. At the same time, the long chip can block the access of the cutting fluid into the cutting area^4^. When the conventional drilling of tough materials, it is not possible to prevent the formation of long chips. The solution can be ultrasonic drilling technology, where vibrations at the frequency of ultrasound are introduced into the classic drilling process. Zhu et al.^16^ focused on drilling the 0.5 mm diameter holes in nickel superalloy with the ultrasound technology. The vibration added to the drilling process broke the chip into small pieces and the high-density energy input disrupts the material under the tool edge resulting in lower feed force.

The influence of the point angle setting on the achieved surface roughness of the drilled hole varies in individual studies. In the case of the studies^8,11^, they observed an increase in surface roughness when the value of the point angle increased, but contrary in studies^10,15^ the authors presented a decrease in surface roughness when the point angle increased. In the study^16^, authors found that the surface roughness decreased about 20–30% when implementing ultrasonic drilling technology.

Accurate drilling of the tool is a prerequisite to produce a high-quality hole, when drilling off-axis, the diameter of the entrance part of the hole increases and the geometric tolerances of the hole deteriorate. The material with lower hardness deteriorates the stabilization of the drill in the drilled hole. Üllen et al.^17^ examined an effect of material structure on the quality of drilled holes when drilling low-alloy chromium-molybdenum steel subjected to different heat treatments. The monitored hole quality parameters were circularity, cylindricality, deviation of the diameter from the nominal value and roughness of the machined surface. It was found that the quality of drilled holes depends on the hardness of the material. When the hardness of the drilled material increased from 34 to 67 HRC (by applied heat treatment), there was an improvement of each individual parameter in the range 30–50%, but conversely, when the hardness of the material decreased from 34 to 24 HRC (by applied annealing), the decrease in hole quality of more than 15% was detected^17^. Improving the ability to drill into soft material can be achieved by implementing ultrasonic drilling technology. In the experiment (carried out by^16^) there was the decrease in the deviation from the nominal diameter from 25 to 5 µm and the conicity of the hole more than 30%.

The main aim of this paper is to study an effect of the different drill geometry (especially the point and clearance angle) on the quality of drilled holes. The quality of drilled holes was assessed by the surface roughness, cylindricity, roundness, perpendicularity of the hole axis, the diameters and position of the individual holes and their mutual comparison. The cutting forces were measured during the drilling operation to verify the effect of different drill geometry. The influence of the tested material surface hardness was evaluated. The novelty of present work is a special design of tested drills, which feature a thin core corresponding with the possibility to set the higher cutting speed with respect to the final hole quality. The thin core utilises to produce a deeper helical groove, which provides the better access for the grinding wheel enabling higher productivity of HSS drills.

## Experimental setup and material

### Experimental material

**Figure 1:**
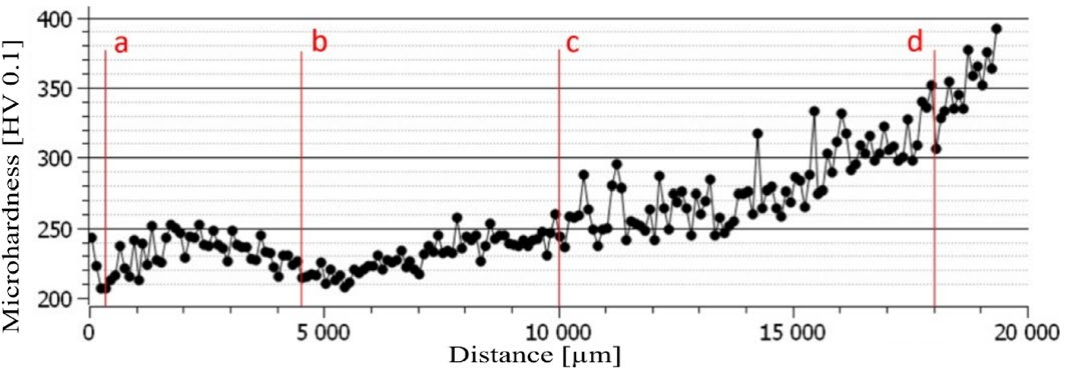
Material microhardness distribution on the cross-section of the drilled material.

The material for drilling was the AISI 304 austenitic stainless steel. The material was in the form of rolled plates with dimensions of 206 × 120 × 20 mm, which were used for the experiment. The drilling operations were carried out into the solid material (without the centering of the hole). The microhardness was measured on both sites of the tested material, which featured the significant difference on the bottom and top site. The microhardness (the course of the microhardness across the material) was observed on the cross-section of the material, in which the microhardness exhibited the range 200–400 HV 0.1 (Fig. 1).

**Figure 2:**
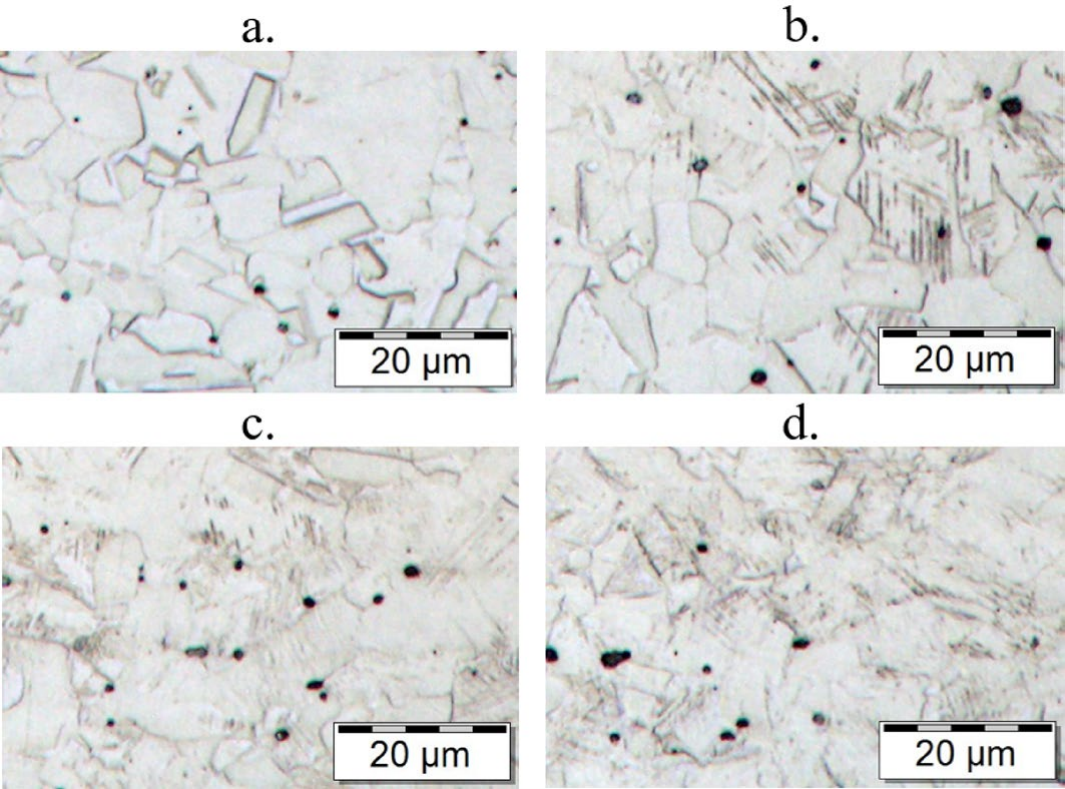
Microstructure of austenitic stainless steel in individual sections of the cross-section.

In order to clarify the cause of the change in hardness, the structure observation was carried out. The position of individual microhardness measurements and microstructure were realised on the cross-section of the material marked in Fig. 2a–d.

On the first side of the plate, the microstructure consists of austenite (Fig. 2a), but towards to the second side of the plate the needles of martensite appeared in the structure (Fig. 2b,c). The martensitic structure was found on the edge of the semi-finished product in the place where the microhardness reached 400 HV 0.1 (Fig. 2d).

The formation of a martensitic structure in the semi-finished austenitic steel was apparently caused by uneven strain hardening during sheet rolling. The side with the higher microhardness exhibited a higher degree of sheet rolling deformation. The similar effect of the formation of the martensitic structure connected with the increase in the microhardness caused by strain hardening during rolling technology was also observed in the study^18^.

### Drilling tools

**Figure 3:**
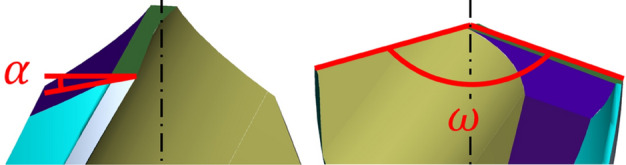
Drill geometry used in the experiment with the clearance angle α and point angle ω.

For the experiment, 8 drills (model CZ005) manufactured by the company NÁSTROJE CZ, s.r.o. were used. The drills were made of M35 high-speed steel with different point angle and clearance angle on the main cutting edge (Fig. 3). These angles were used to verify the influence of the drill geometry during the drilling process.

The point angle for individual drills was in range 135°–145° and the clearance angle was in range 7° to 15° (see Table 1). The range of a clearance angle was selected as a minimum and maximum value (and values within the range), which can be grinded with the grinding wheel and the point angle was chosen as the similar angle used for sintered carbide drill production Each drill (A–G) was produced twice due to verification of their repeatability.

**Table 1:** The values of the point angle and the clearance angle for the individual compared drills.

	A	B	C	D	E	F	G
Point angle ω (°)	135.68	135.62	138.32	138.35	141.61	142.14	145.03
Clearance angle α (°)	8.09	7.20	6.92	7.07	7.76	8.37	14.65

The geometry of the drill was based on the CZ005 model series of drills designed for machining stainless steel. The specifications of the drills used in the experiment (see Table 2) were the same for all the tested samples.

**Table 2:** Specifications of experimentally used drills.

Drill diameter	13 mm	Helix angle	29.40 ± 0.40°
Overall drill length	151 mm	Clearance angle in the axis of the drill	1.70 ± 0.80°
Flute length	101 mm	Drill core thickness	1.00 ± 0.10 mm

**Figure 4:**
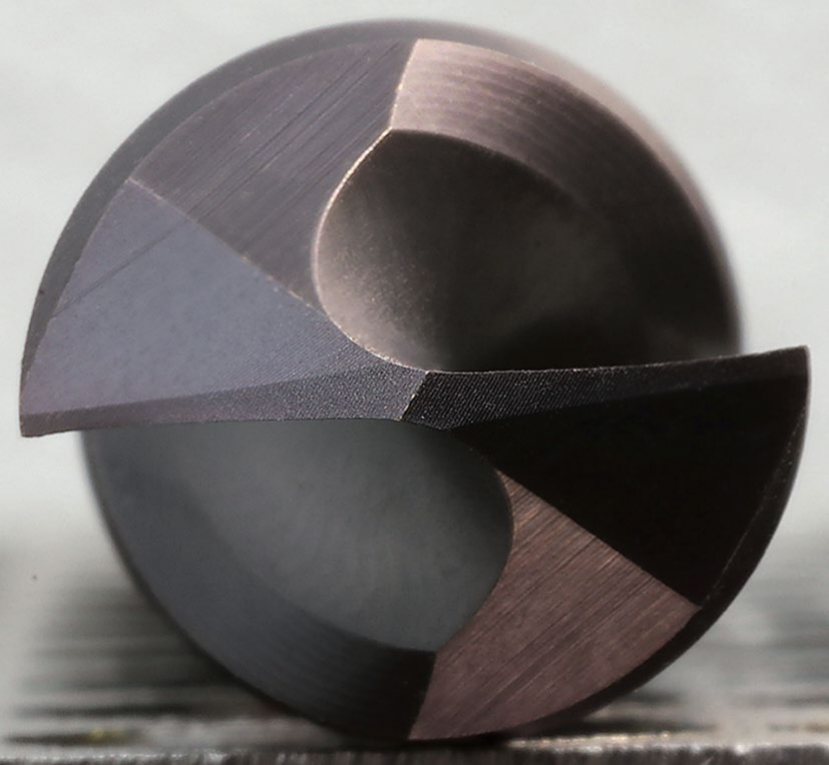
Detail geometry of used drill CZ005.

The geometry of individual drills was measured on a HELICHECK PLUS optical measuring machine from the company Walter. The geometry of the CZ005 drill is particularly characterized by a very thin core, where the diameter of the core is 0.07–0.08 × D. Due to the thin core, it is possible to produce a deeper helical groove in the drill. The deeper groove the more positive geometry can be grinded (see Fig. 4). The dependence of clearance angle on the depth of the groove is given by the grinding process. The deeper groove the bigger space for the grinding wheel enabling better grinding process of the cutting edge^19^.

The positive clearance angle along the entire length of the edge plays a significant role in case of the machining austenitic stainless steel. A positive clearance angle of the main cutting edge reduces the noneffectual zone within the area in the vicinity of the drill axis. With this geometry it is possible to reach the reduction of the cutting forces, deformation hardening and heat generation within the area of the drill tip. These aspects are considered to cause a poor machinability of austenitic stainless steel^20^. The reduction of the core cross-section at the point of the drill causes a significant reduction feed force^21^.

The clearance of the drill CZ005 is created by three individual facets depicted in Fig. 4, while the facet along the main edge features the original shape. The second edge exhibits the facet with its width corresponding with the drill core diameter. The facet is decreasing towards to the outer perimeter of the drill to cause the lower friction between the clearance and the bottom of the drilling hole^19^.

The shape of the main edge is created from the 2/3 by the convex curve, which is followed with the flat area of the main edge corresponding to the rest of 1/3 of the main edge^19^.

### Experimental and measurement design

**Figure 5:**
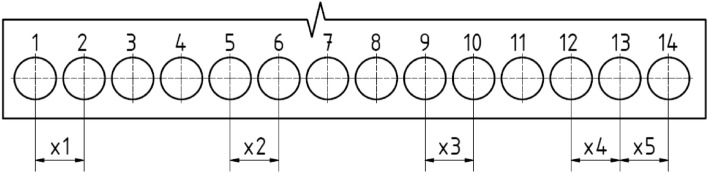
Scheme of the experimentally drilled holes on the drilled plate.

Testing of the individual listed drills was carried out on the multifunctional vertical machining centre TAJMAC ZPS MCV 1210 (BUT, Czech Republic, Brno). Total of 14 holes (see Fig. 5) were drilled with each of the mentioned tools as part of the experiment. The number of 14 holes was set to evaluate the geometrical tolerances and find out how they change during the drilling (from the 1st to 14th hole). The drill was not worn after the 14th hole, but the test was ended (the necessary data for evaluation of the proper tool geometry were obtained).

The drilled holes were analysed considering the following factors:

- cutting forces (1st and 14th hole),
- roughness (1st and 14th hole),
- geometric tolerances—circularity, cylindricality, and perpendicularity of the axis of the hole to the surface of the plate (1, 2, 5, 6, 9, 10, 12, 13 and 14 hole),—diameter (1, 2, 5, 6, 9, 10, 12, 13 and 14 holes),
- pitch (x1, x2, x3, x4, x5).

#### Cutting forces measurement

A KISTLER 9272 dynamometer (Feed force in range − 5 to 5 kN < 0.02, torque in range − 200 to 200 < 0.2 Nm) with accessories was used for the purpose of the experiment to measure the cutting forces. The dynamometer was clamped to the worktable of the machining centre using T-slot screws, a vice was attached to the dynamometer for clamping the drilled plate. Cutting forces were measured only when drilling into the side corresponding with the higher observed microhardness (400 HV). The cutting forces were affected only by the change in the geometry of the drill, the other parameters of the process were constant during the experiment.

#### Surface roughness measurement

**Figure 6:**
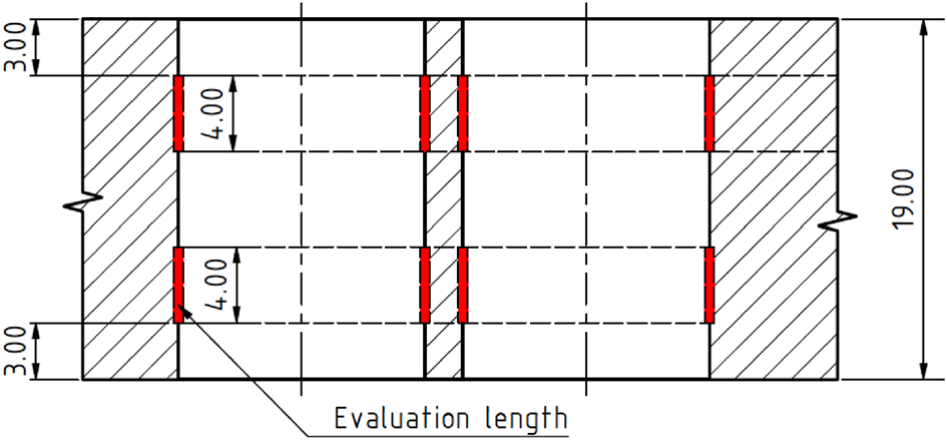
Methodology used for the measuring of the surface roughness of drilled holes.

The surface roughness of the holes was measured using a Taylor Hobson Talysurf CLI 1000 roughness device. According to the ČSN EN ISO 4288 standard, the indicative value of the RSm parameter was first measured and the corresponding basic and evaluated length was chosen based on the measured values. After levelling the surface to a horizontal position, the profile of the entire length of the hole was measured. To obtain the roughness profile, it was necessary to perform thresholding, remove the shape of the measured profile using a 5th-order polynomial, and filter the measured data using a Gaussian filter. The evaluated lengths were extracted from the obtained roughness profile (see Fig. 6), and the required roughness parameters Ra and Rz were subsequently measured.

#### Geometric tolerances measurement

**Figure 7:**
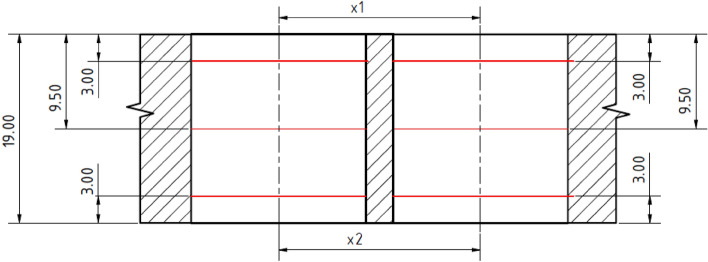
Methodology used for the measuring of the tolerances of the geometry of drilled holes.

Measurements of geometric tolerances i.e., hole diameters and pitches were performed on the Leitz Reference Xi coordinate measuring device. The circularity, the perpendicularity of the hole axis to the surface and the spacing of the holes were measured in two places along the length of the hole in places 3 mm from both sides of the material (see Fig. 7). Roundness was evaluated based on the centre of the least squares according to the ČSN ISO 4291 standard. The spacing of the pair of holes is considered as the spacing of a pair of circles at the same height level created during the roundness evaluation. The tolerance field of the hole perpendicularity is considered as the cylinder in which the axis of the cylinder of the approximated hole falls.

The evaluation of the cylindricity and diameter of the drilled holes took place in three places along the length of the holes (3 mm from the top of the hole, the middle height and 3 mm from the bottom of the hole, the scheme is depicted in Fig. 7). The cylindricity of the holes was determined based on the creation of the so-called wrapping cylindrical surfaces created by extracting the circularity profile according to the ČSN ISO 12180-2 standard. The diameter of the holes is considered as the diameter of a circle approximated by the method of least squares.

#### Determining the accuracy of the position and dimension of the holes

The position of the holes was assessed based on the measurement of the deviation of the pair of holes from the nominal value of the spacing. In the case of the dimension, the parameter was the deviation from the nominal value of the diameter.

### Cutting conditions

The cutting conditions were set to the indicated values and remained constant throughout the experiment i.e., cutting speed v_c_ = 32 m min^−1^ (corresponding revolutions n = 784 min^−1^) and feed per rotation f = 0.2 mm (corresponding feed rate v_f_ = 157 mm min^−1^). Quaker Cool 7350BFF cutting fluid with a concentration of 5% (recommended by the producer of the drills) was used during drilling.

## Results and discussion

**Figure 8:**
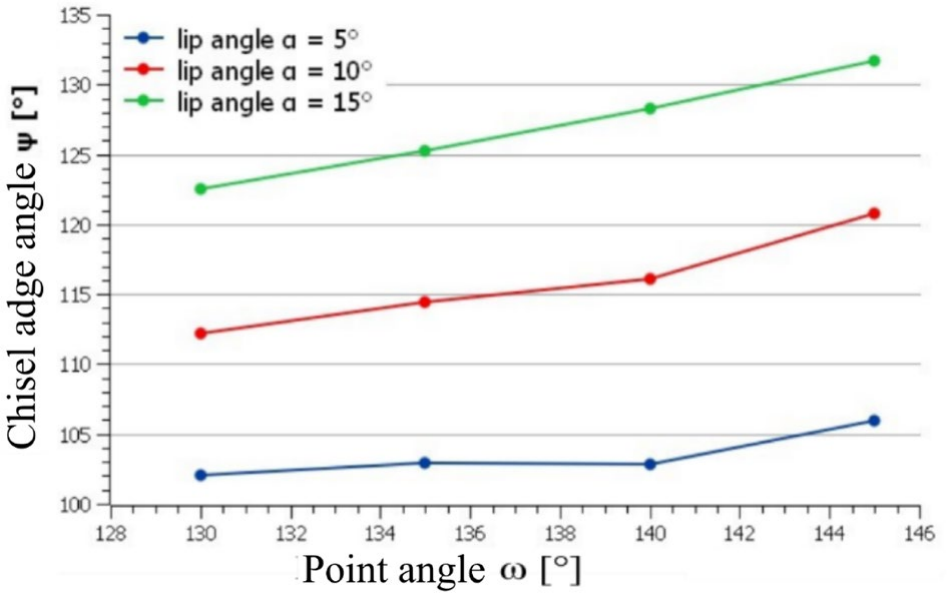
Dependence of the angle size of the cross blade on the setting the value of the point angle and the clearance angle of the drill CZ005.

The following chapter presents the obtained results based on observing the influence of the point angle and the clearance angle on the quality of the drilled holes. Using the simulation in the Helitronic tool studio program, the effect of both mentioned angles on the size of the angle of the cross edge of the drill was determined (see Fig. 8).

### Feed force and torque evaluation

**Figure 9:**
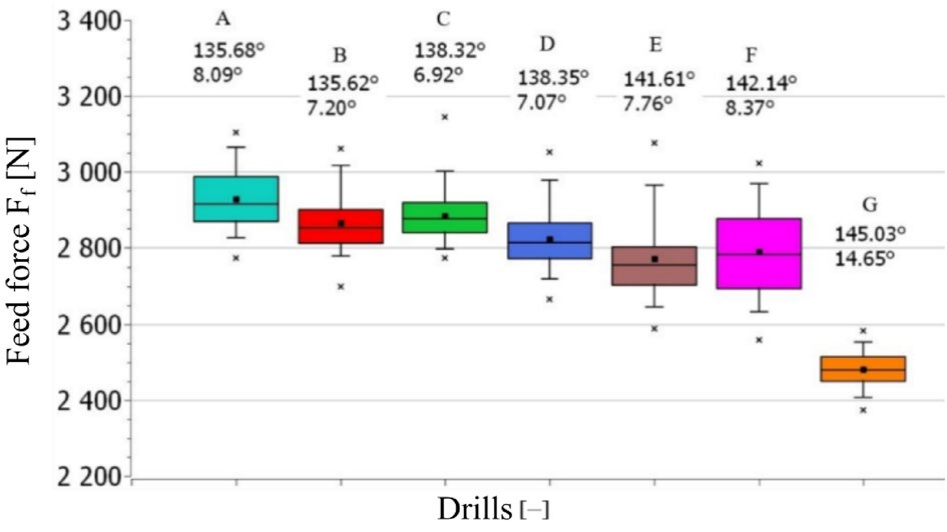
The influence of the drill bit geometry on the magnitude of the applied feed force.

**Figure 10:**
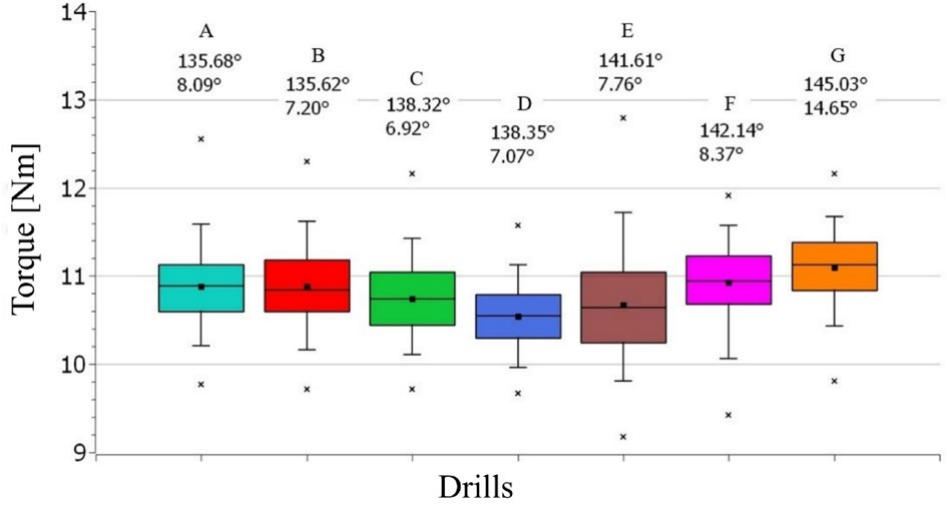
The influence of the drill geometry on the applied torque magnitude.

The feed force and torque were measured for individual drills used in the experiment. The Figs. 9 and 10 are depicted for the drilling of 1st hole (new tool). The boxplot chart was used to visualize the data, due to the possibility to display the aggregated statistical data. Individual blocks represent 50% of the data set, falling between the 1st and 3rd quartiles. The lines leading from the blocks show the distribution of the rest of the values of the set, the outliers are expressed by separate points. The straight line inside the block represents the median of the set and the black block the mean value of the data set. The average value of the feed force when drilling with individual drills was in the ranged 2481–2929 N (see Fig. 9).

Based on the acquired data, it can be stated that point and clearance angles influenced the applied feed force (compare drill A and drill G, where the maximum difference 420 N was observed). When the clearance angle increased by less than 1° in drills A and B, the feed force decreased more than 70 N. The example of the influence of the size of the point angle can be observed in drills A and E, where the point angle increased by 6°, the feed force decreased more than 150 N (see Fig. 9). An increase of the clearance angle (from 8.37° correlates with drill F to 14.65°corresponds with drill G) caused a decrease (270 N, calculated from average values) of the feed force, which can be imparted by the creation of a sharper cutting edge resulting in lower resistance against penetration into the workpiece material. The torque was less affected by the change of angles compared to the feed force. The torque value varied between 10.5 and 11.1 Nm, depending on the selected value of the clearance angle and point angle (see Fig. 10). The highest torque value was measured in case of drill G, which is characterized by the highest value of both angles (point and clearance angle).

### Hole surface roughness

**Figure 11:**
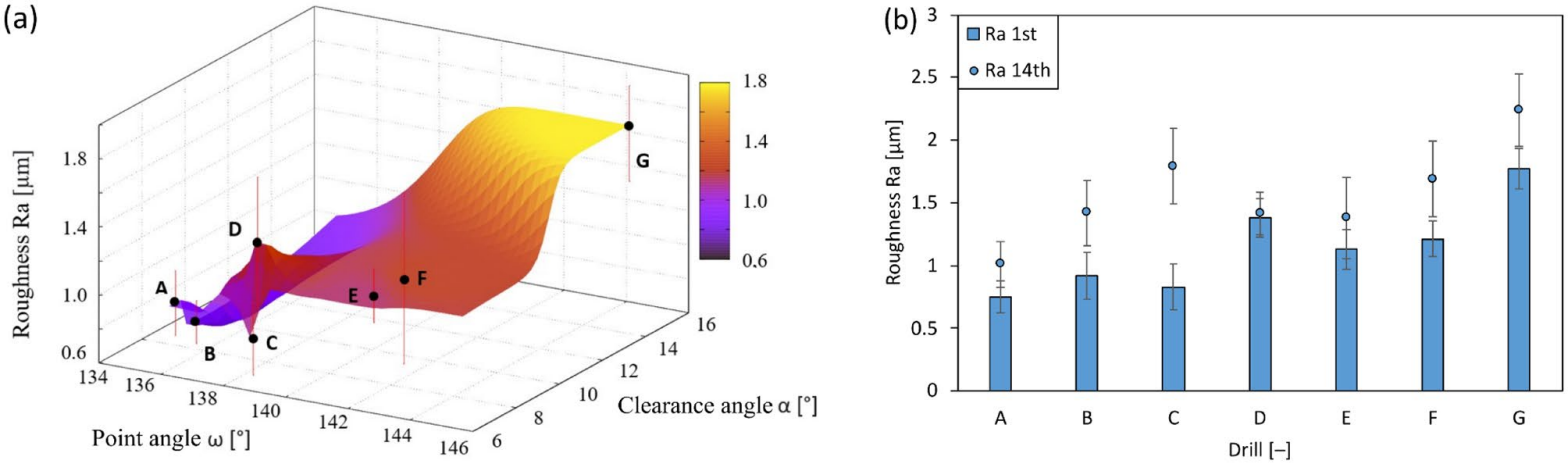
(**a**) The dependence of the deviation value from the nominal value of the point angle and the clearance angle of the drill on the achieved surface roughness and (**b**) roughness for the 1st and the 14th hole reached for each drill.

The surface roughness of the drilled holes was evaluated based on the parameters Ra and Rz, the measurement was carried out according to the methodology described in chapter 2.3.1. Using the lower values of both observed angles resulted in surface roughness lower than 1 µm considering three compared drills i.e., A, B and C. The dependence of surface roughness on the point and clearance angle is depicted in Fig. 11a. Drill F exhibited the highest deviation (represented by the red line depicted in each drill) in regards of roughness Ra, but contrary the lowest deviation featured drill B with the lowest point angle (132.62°). Figure 11b shows roughness for the 1st and the 14th hole reached for each drill.

The observed dependence of surface roughness on the point and clearance angles, showed the same trend in case of Rz parameter, therefore the display of the measured values of Rz was not performed. The surface roughness parameter Rz was evaluated in the range 5.7–11.7 µm. The influence of the angles on the surface roughness is based on the premise to maintain a smooth machining process without introducing additional vibrations and uneven force stress. The drill geometry with the lower clearance angle shows the sharper cutting wedge and makes it easier for the tool to cut into the workpiece material. Choosing a lower point angle influences the ability to stabilize the drill in the hole due to more favourable force action. When both angles described above are changed, the emerging phenomena interact with each other. In the case of choosing a lower value of the angle of the point and the clearance angle (in case of A and B drills, see Fig. 11), the drill is sufficiently stable when cutting into the material. In the case of the higher value of the point and clearance angle the cutting edge repeatedly cuts into the workpiece material and then cause undesirable vibrations. The described effect resulted in higher surface roughness noticeable in case of drills E, F and G.

### Geometric tolerances of holes

**Figure 12:**
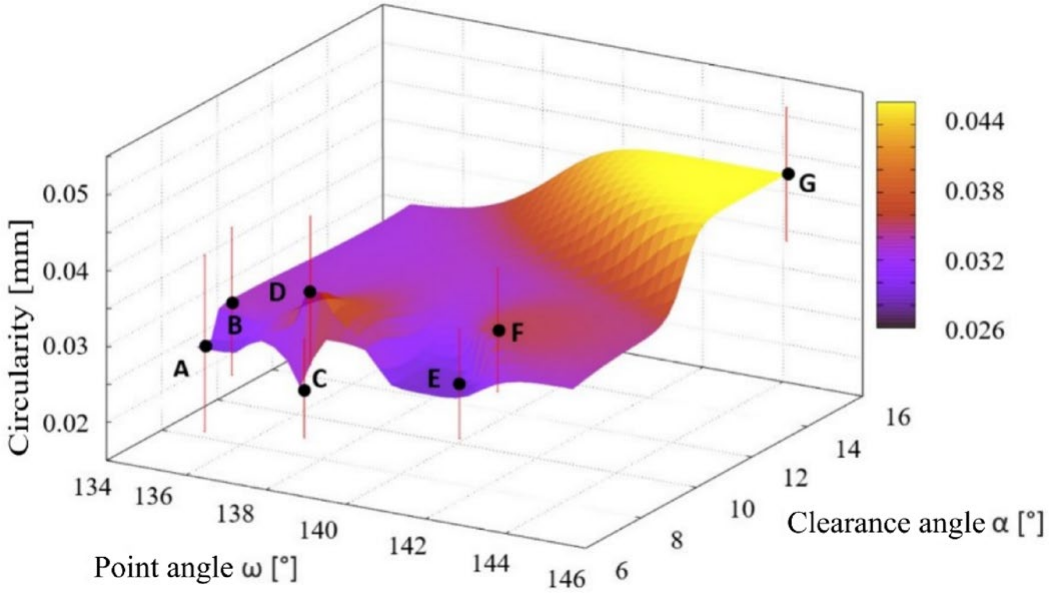
The dependence of the deviation value from the nominal value of the circularity value of the drilled holes on the choice of point angle and clearance angle.

**Figure 13:**
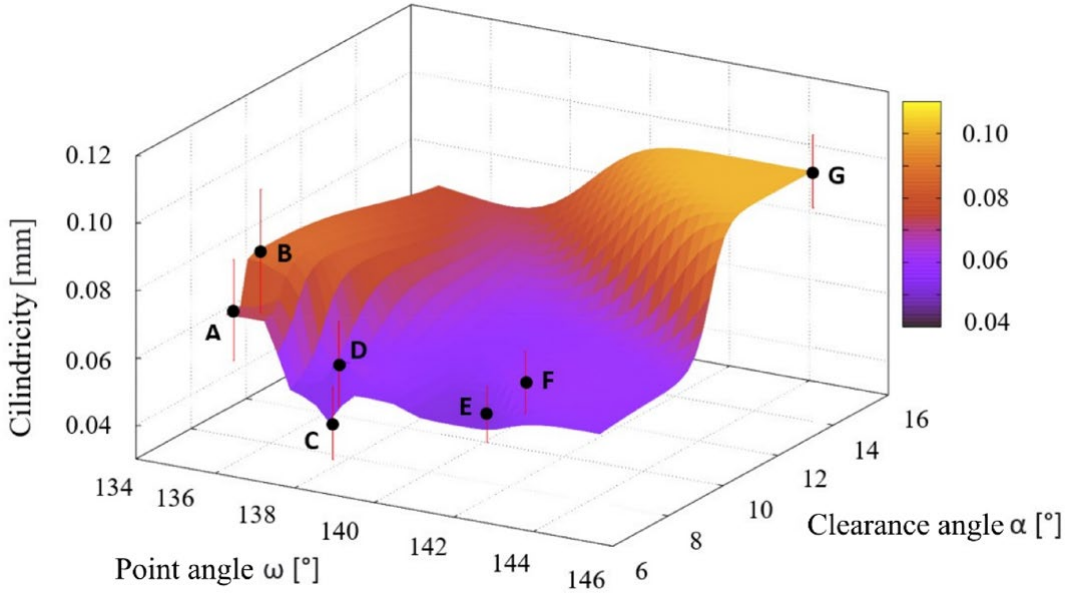
The dependence of the deviation value from the nominal value of the point angle and the clearance angle of the drill on the size of the drilled hole cylindricity.

**Figure 14:**
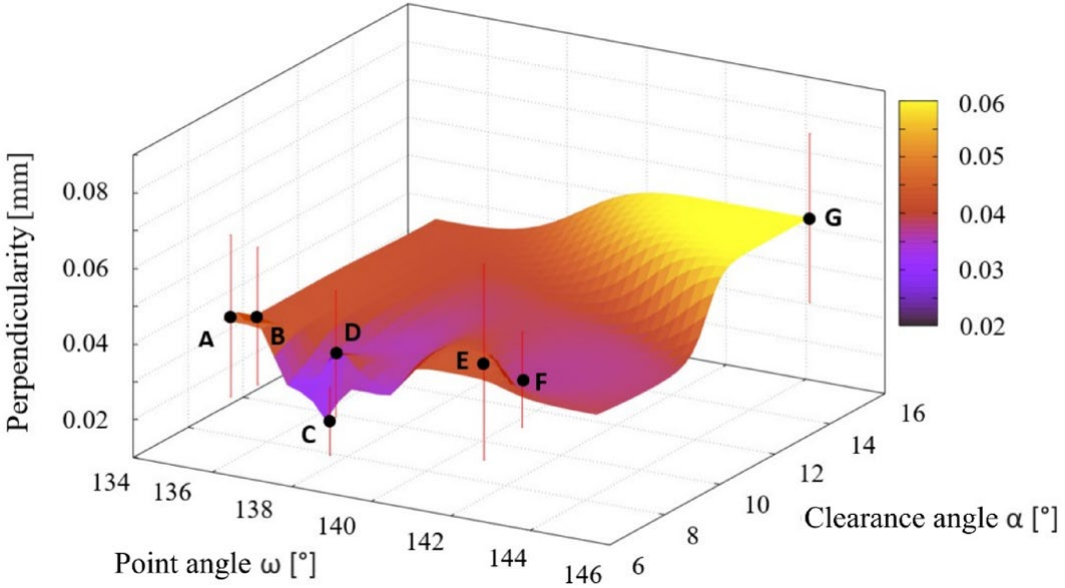
The dependence of the deviation value from the nominal value of the chosen geometry of the compared angles on the achieved perpendicularity of the axis of the hole to the surface of the drilled plate.

The geometric shape was assessed using the measured geometric tolerances of the holes—circularity (see Fig. 12), cylindricity (see Fig. 13) and perpendicularity of the hole axis to the surface (see Fig. 14). The lowest values of geometric tolerances were measured in case of the drill C, but contrary the highest values of geometric tolerances were found in case of unstable drill G.

The measured values of the geometrical tolerance (calculated as an average value from holes described in “Experimental and measurement design” section) of roundness ranged from 0.025 (drill C) to 0.045 mm (drill G). Changing the angle of the point and the clearance angle on the main edge for drills A to F featured no effect on the roundness of the measured holes. A significant effect of increasing the clearance angle was found in case of drill G (Fig. 12). Drill A exhibited the highest deviation considering a hole circularity, but contrary the lowest deviation featured drill C with the lowest clearance angle (6.92°).

The cylindricity of the analysed holes (calculated as an average value from holes described in “Experimental and measurement design” section) was measured in a range of values from 0.045 (drill C) to more than 0.095 mm (drill G). Higher cylindricity values were reached when using drills with point angle values of 138°–142° and clearance angle values from 7° to 8° (Fig. 13). Drill B exhibited the highest deviation considering a hole cylindricity, but contrary the lowest deviation showed drill E (point angle 141.61 and clearance angle 7.76° respectively).

The measured values of the perpendicularity (calculated as an average value from holes described in “Experimental and measurement design” section) of the hole axis to the surface ranged from 0.025 (drill C) to 0.06 mm (drill G). The best values were obtained using a point angle in the range of 138°–140° (Fig. 14). Drill E featured the highest deviation considering a hole perpendicularity (opposite to hole cylindricity), but contrary the lowest deviation featured drill C as in case of hole cylindricity.

Drill G achieved significantly worse hole quality results in terms of surface roughness and geometric tolerances compared to drill D with a very similar geometry. The cause of the described anomaly is apparently a defect in the drill caused during its production i.e., elevation of the blade, when the outer tips of the drill have a different height. This defect causes a different engagement of the two edges of the tool, resulting in the vibrations and regular misalignment of the drill bit during drilling. The achieved values of geometric tolerances are mainly influenced by the ability to accurately drill into the material and the subsequent stability of the cutting process during drilling. Improper entering of the drill into the material can impart the axis of the hole to deviate and introduce vibrations.

### Position and size of holes

**Figure 15:**
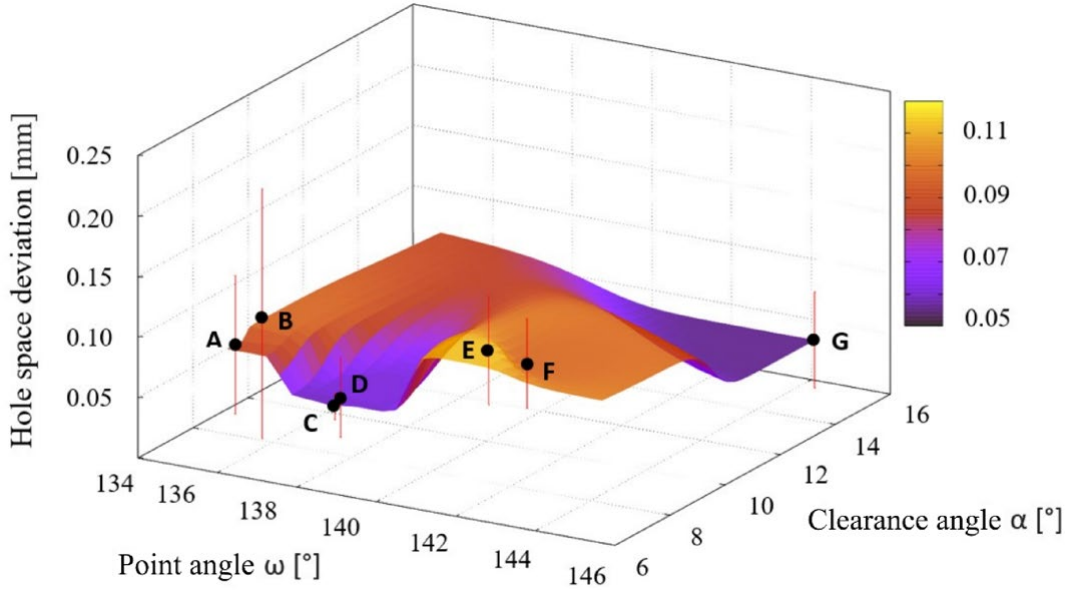
The dependence of the deviation value from the nominal value of the drilled holes spacing on the choice of the point angle and the clearance angle on the drill.

The values of the deviation from the nominal value of the hole pitch were for individual holes from 0.06 mm (in case of drill C) to 0.1 for drill E (see Fig. 15). The best values were achieved with drills C and G. In the case of hole machined by drill C, this value was assumed as the high-quality drilled holes. The value of the drill G hole is not considered due to the very poor quality of the hole in terms of assessed parameters. The standard deviation of the set of measured data for pitch deviations from the nominal value is only 0.01 mm, this fact confirms the stability of the drill during drilling into the material. Drill B featured the highest deviation considering a hole space deviation, but the lowest deviation value was reached by drill C.

**Figure 16:**
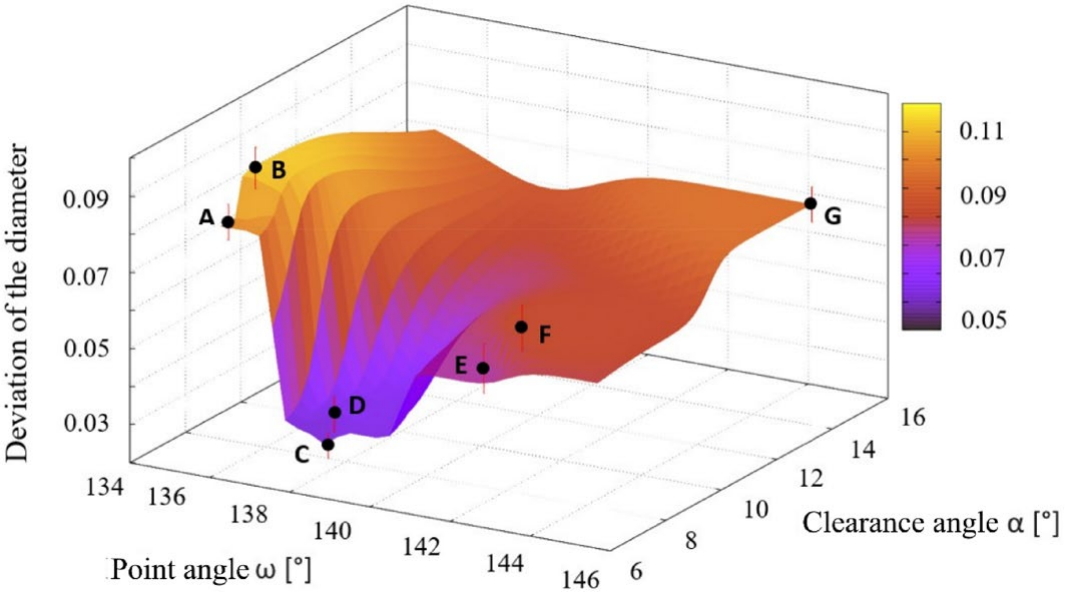
The dependence of the deviation value from the nominal value of the point angle and the clearance angle of the drill on the size of the deviation from the nominal value of the drilled hole diameter.

In terms of the accuracy of the dimension of the drilled holes, the best results were found for drills C and D, with a point angle of 138° and the clearance angle of 7° (see Fig. 16). The deviation values of the measured data from the nominal value of the diameter are 0.03 mm and 0.036 mm respectively for the mentioned drills, and the results for other drills are two or three times higher. The lowest deviation was performed by drill C as in previous cases.

The correlation between the measured values of the deviation of the pitch and the diameter from the nominal value for all compared drills except tool G was found. Drilling out of the correct position affects the accuracy of the hole dimension due to the creation of a radial force. In the entrance part of the hole, this effect manifests itself more intensively, because there is no stabilization of the drill bit in the hole (see Table 3).

**Table 3:** Comparison of the difference in the diameter value in the inlet and outlet parts of the drilled hole.

Drill	A	B	C	D	E	F	G
d_inlet_ – d_outlet_	0.01940	0.01051	0.00051	0.01508	0.02929	0.01709	0.04690

From the variable parameters, the angle of the cross blade featured the highest influence on the accuracy of the position and size of the hole, because it is the first part of the drill in contact with the workpiece material. In the case of drills with an inadequate geometry of the cross blade, unstable drilling occurs when drilling, the so-called dancing of the drill.

### Influence of the material hardness

**Figure 17:**
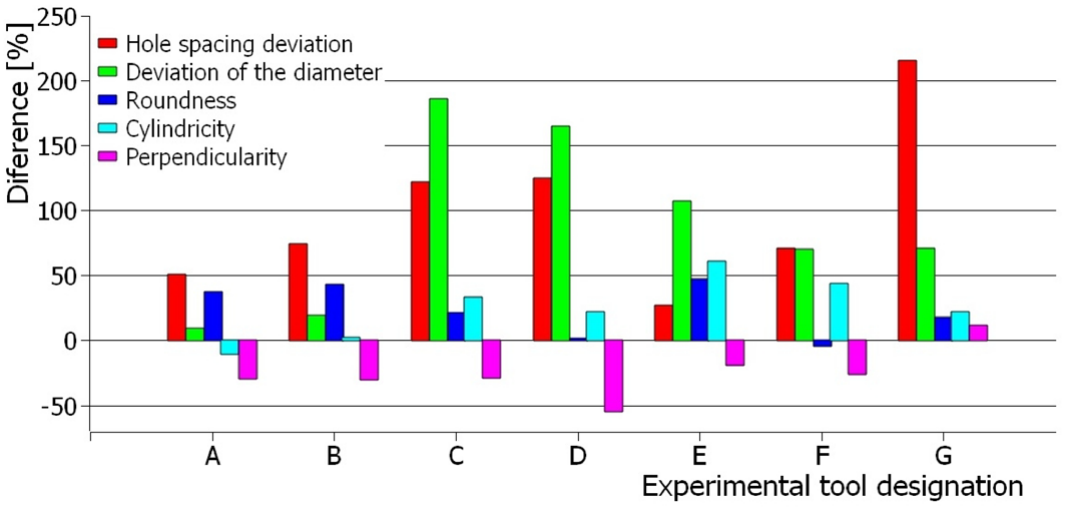
The influence of the surface drilled material hardness on the individual parameters of the hole quality.

Drilling operation was done into material with different cross-sectional microhardness corresponding with the Fig. 1. In the first case, the lead in of the drill was into harden material and the lead out was performed into soft material. The material featured the surface microhardness of 200 HV0.1 and 400 HV0.1 on the soft and the harder site of the material respectively. The goal of the experiment was to analyse the effect of microhardness on the quality of drilled holes. It was found that when drilling into soft material, the quality of the drilled holes deteriorated in terms of all parameters except the perpendicularity of the hole. Individual columns for drills A to G show the percentage reduction in quality according to individual criteria (see Fig. 17). A decrease in hole quality was caused by a decrease in the CZ005 drill's ability to accurately drill into soft material. A layer of harden material on the surface with a thickness of about 5 mm worked similarly to a drill sleeve (lead the drill) and prevented the tool from misaligning during drilling. The microhardness of the material when the drill leads out the hole did not affect the quality of the holes, because the tool was already stabilized in the created hole even in case of soft material.

The most significant influence of the microhardness of the surface layer of the drilled material was observed in the accuracy of the diameter and spacing of individual holes. At the same time, it was found that the microhardness of the material featured the different effect on the individual geometries of the tool. For drills C and D, the decrease in diameter and pitch accuracy of more than 100% was measured. The reduction of the hardness of the surface layer of the material had the least effect on the quality of the holes in the case of the drill with the lowest point angle.

### Chip morphology

For individual drills, the shape of the formed chips was monitored in the experiment. Correct chip formation is important in drilling for the rapid removal of chips from the cutting area and the prevention of damage to the surface of the hole by outgoing chips. From the morphology of the chips, it is possible to retrospectively evaluate the drilling process.

**Figure 18:**
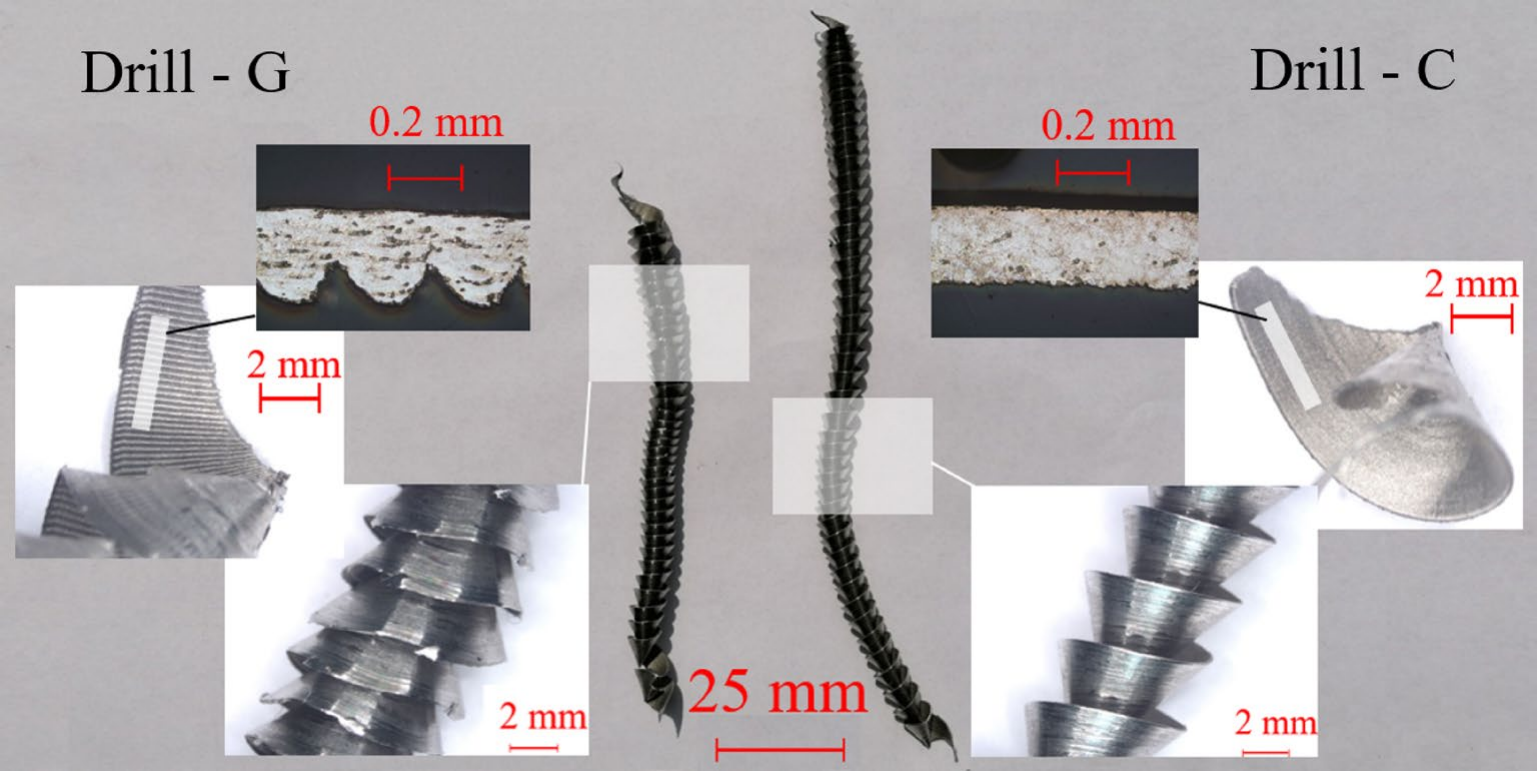
Comparison of the chip morphology formed by the stable drill C and the chips formed during the unstable drilling process by the tool G.

The shape of the chips formed by drills A to F featured no significant difference due to the maintenance of the stability of the solid drills. In case of drill G, there was a significant deformation of the ideal shape of the chip, the formation of notches on the edge of the chip and the formation of an uneven cross-section along the length of the chip (see Fig. 18). The shape of the resulting chip (drill G) was affected by the instability of the process described in chapter 3.1.

## Discussion

When the point angle value of the CZ005 drill bit increased in the experiment, the feed force decreased. Some studies confirmed this conclusion^10,11^, however, the opposite dependence was presented in the studies^6,12,13^. When comparing the conditions of the individual experiments, it was found that the feed force decreased when the value of the point angle increased in the process of drilling into pre-drilled holes^10,11^, where the cross-cutting edge of the tool is not in contact with the workpiece. The CZ005 drill achieved a similar dependence due to the very thin core at the point of the tool. When considering the above hypothesis, it is possible to state that in the case of drills with a normal core thickness and a high value of the point angle, an unsatisfactory material forming process occurred when drilling into solid material at the point of the cross edge. As a result of the inefficient machining process in the vicinity of the axis of the drill, there was the increase in the feed force.

It was found that the pair of point angle and clearance angle affects the stability of the drill bit in the hole. The stability of the drilling process directly determines the achieved surface roughness and the shape of the chip formed. Increasing the point angle in the range of 135° to 142° featured no significant effect on the stability of the process. As a result of a significant increase in the angle of the clearance in the drill G, the stability of the drilling process was reduced. This process was evaluated using the change in roughness and shape of the formed chip. The effect of the pair of point angle and clearance angle is apparently interconnected. The clearance angle determines the angle of the cutting wedge, increasing the angle of the clearance creates a sharper cutting geometry. The sharp cutting geometry cuts the workpiece material more easily. Lower point angle values stabilize the drill bit in the hole^11^. Based on the mentioned hypothesis, it can be concluded that a sharper cutting geometry featured the ability of stable machining under the action of lower cutting forces, if the bit is stabilized using a lower value of the point angle. In this case, the drilled hole shows a low surface roughness (see Fig. 11) and a uniform chip without a notch and deformation is formed (see Fig. 18). In the case of using a sharp cutting geometry and a drill with a higher value of the point angle, the cutting edge will cut the material and then jump out of the cut due to the low stabilization of the drill. This process introduced vibrations into the cut and the result is a significant deterioration of the surface roughness and a poorly formed chip.

The chosen value of the clearance angle and point angle according to the measured data affects the geometric accuracy of the drilled holes. The geometric accuracy of the holes is affected by the choice of the given angles, apparently indirectly through the dependence of the clearance angle and point angle on the size of the cross blade angle on the drill. Accurate and stable drilling into the material is a prerequisite for drilling a geometrically accurate hole^16,17^. The size of the angle of the cross blade is important for stable drilling because the cross blade is the first part of the drill with the material to be drilled, and the tilt of the cross blade determines the direction of the resulting cutting forces when drilling the tool.

The geometric accuracy of the drilled holes in the experiment depends on the hardness of the material and the surface of the drilled plate. When the hardness of the drilled material was reduced, the quality of the holes was reduced in terms of geometric tolerances, the deviation of the hole spacing and the deviation from the nominal diameter. This conclusion is consistent with the results of the mentioned studies^17^. When drilling into the material, as a result, vibrations will be created in the sudden need to overcome the resistance of the material. In the case of drilling soft material, the cutting drill bit will bounce slightly and subsequently drill outside the axis of the drill. The solution to reduce vibrations and improve the drilling ability would probably be to reduce the feed rate when drilling. Additional forces act in the direction of the original axis of the drill, and the drilled hole has a characteristically higher diameter in the entrance part of the hole and a conical shape^16^. A layer of the material with a higher hardness, or a material with an overall higher hardness, influences the principle of the drill sleeve and thus prevents the drill bit from being deviated from the correct axis of the hole.

## Conclusion

Drills marked CZ005 made of M35 high-speed steel with different geometry were subjected to cutting tests when drilling into solid material. The cutting forces, surface roughness, geometric tolerances, spacing and diameter deviations were compared to the nominal values of the drilled holes. Based on the analysis of the measured results, the following conclusions were drawn:

- When the size of the point angle and the clearance angle increased, the feed force decreased. The increase of the point angle by 6° resulted in the decrease in the feed force of more than 150 N. In the case of the increase in the value of the clearance angle by 1°, the decrease in the feed force of 70 N was observed.
- The surface roughness according to the Ra and Rz parameters was lower than 1 µm and 6 µm respectively. The best surface roughness was achieved with the tool with the lowest pair of point angle and clearance angle values. The increase in both compared angles resulted in the increase of roughness up to the value of Ra 2.24 µm (Rz 11.7 µm).
- The best results in terms of geometric tolerances of roundness (0.025 mm), cylindricity (0.045 mm) and perpendicularity of the hole axis (0.025), deviation from the nominal value of the spacing (0.06 mm) and deviation from the nominal value of the diameter of the hole (0.03 mm) was achieved with the C drill.
- For drilled holes, the inlet diameter of the hole was higher than the outlet diameter due to unstable drilling. The lowest difference between the inlet and outlet diameter of the hole was found for drill C, when the value of the difference was only 0.0005. For holes created by other drills, the values of this parameter were up to 0.047 mm.
- Reducing the hardness of the material on the surface of the drilled plate from 400 HV0.1 to the half value resulted in the deterioration of the geometric accuracy of the drilled holes by up to 220%, depending on the individual parameters.
- The range of recommended values for the point angle is 138°–142°, the clearance angle 7°–10°.

The performed research helped to obtain a new design of the drill tool, which is cheaper in price (compared to the sintered carbide tool) and can be used for higher cutting speed (32 m min^−1^) in comparison to standard produced HSS drill tool.

## Data Availability

The datasets used and/or analysed during the current study available from the corresponding author on reasonable request.
